# Biologics in Older Adults with Chronic Obstructive Pulmonary Disease: A Narrative Review

**DOI:** 10.3390/jcm15166277

**Published:** 2026-08-13

**Authors:** Simone Scarlata, Alessio Marinelli, Panaiotis Finamore, Vitaliano Nicola Quaranta, Giulia Amoroso, Alessandra Tomasello, Claudio Sorino, Giovanna Elisiana Carpagnano, Nicola Scichilone, Silvano Dragonieri

**Affiliations:** 1 Unit of Respiratory Pathophysiology, Department of Internal Medicine and Geriatrics, Campus Bio Medico University and Teaching Hospital, 00128 Rome, Italy; 2 Institute of Respiratory Disease, Department of Traslational Biomedicine and Applied Neuroscience, University of Bari “Aldo Moro”, 70124 Bari, Italy; alessio.marinelli@uniba.it (A.M.); g.amoroso4@phd.uniba.it (G.A.);; 3 Division of Respiratory Medicine, PROMISE Department, “Paolo Giaccone” University Hospital, 90127 Palermo, Italy; 4 Department of Pulmonology and Cardiorespiratory Rehabilitation, Sant’Antonio Hospital, 22063 Cantu, Italy

**Keywords:** chronic obstructive pulmonary disease, older adults, biologic therapies, immunosenescence, frailty

## Abstract

**Background**: Chronic obstructive pulmonary disease (COPD) disproportionately affects older adults. While targeted biologic therapies have shown efficacy in type 2 eosinophilic endotypes, elderly patients remain underrepresented in pivotal clinical trials. This narrative review examines the pathophysiological rationale, efficacy data, and unique geriatric challenges of biologic therapies in older adults. **Methods**: A literature search was conducted in PubMed and Scopus for phase II and III randomized controlled trials (RCTs) and observational studies evaluating biologic agents (anti-IL-5, anti-IL-4/IL-13, and anti-alarmins) in COPD, focusing on populations aged ≥ 65 years, comorbidities, and frailty. **Discussion**: Phase III data for dupilumab and mepolizumab indicate significant reductions in annualized exacerbations in patients with blood eosinophils ≥ 300 cells/μL. However, the mean age in the landmark trials (65 years) is a decade lower than the real-world average. Age-related immune remodeling—specifically immunosenescence and inflammaging—coexists with disease-specific pathways, potentially altering therapeutic responses. Furthermore, clinical trials systematically exclude older individuals with severe multimorbidity, physical frailty, cognitive impairment, and malnutrition. **Conclusions**: Biologics provide a precise, mechanism-based approach, yet evidence in the oldest-old remains limited. Future management should utilize the treatable traits framework, shifting selection criteria from chronological age to biological age metrics, including frailty status and functional reserve.

## 1. Introduction

Chronic obstructive pulmonary disease (COPD) represents one of the leading causes of morbidity and mortality worldwide, with its burden disproportionately affecting older adults [1]. The prevalence of COPD increases markedly with age, reaching nearly 30% in individuals older than 75 years, while exacerbations, hospitalizations, functional decline, and mortality occur more frequently in older patients [2]. Despite advances in inhaled pharmacotherapy, including triple inhaled therapy with inhaled corticosteroids, long-acting β2-agonists, and long-acting muscarinic antagonists, a substantial proportion of patients continue to experience persistent symptoms and recurrent exacerbations [3]. This residual disease burden highlights a major unmet therapeutic need, particularly among older patients characterized by multimorbidity, polypharmacy, frailty, and accelerated lung function decline. Current treatment strategies remain largely focused on symptom control and exacerbation prevention, while limited options are available to specifically target the underlying inflammatory mechanisms driving disease progression [4].

Recent advances have demonstrated that COPD is biologically heterogeneous and comprises distinct inflammatory endotypes. In particular, a substantial subgroup of patients exhibits evidence of type 2 (T2) inflammation, characterized by eosinophilic airway inflammation and activation of cytokine pathways involving IL-4, IL-5, and IL-13. These findings have provided the rationale for the development of targeted biologic therapies and opened new perspectives for precision medicine in COPD.

Nevertheless, older patients remain markedly underrepresented in pivotal clinical trials, despite constituting the majority of the real-world COPD population [5].

### 1.1. Objectives

The primary objective of this narrative review is to critically evaluate the clinical efficacy, safety, and pathophysiological rationale of targeted biologic therapies in older adults with chronic obstructive pulmonary disease (COPD). The secondary objectives are to identify the empirical evidence gaps caused by the underrepresentation of geriatric populations in landmark clinical trials, examine how immunosenescence and inflammaging interact with type 2 inflammatory pathways, and propose a multidimensional framework incorporating frailty, cognitive function, and nutritional management into future precision prescribing strategies.

### 1.2. Material and Methods

A comprehensive literature search was conducted across the PubMed and Scopus databases up to June 2026. The search strategy utilized combinations of MeSH terms and keywords, including: ‘pulmonary disease, chronic obstructive’, ‘aged’, ‘biologics’, ‘mepolizumab’, ‘benralizumab’, ‘dupilumab’, ‘tezepelumab’, ‘frailty’, and ‘immunosenescence’. Selection criteria were restricted to peer-reviewed phase II and phase III randomized controlled trials (RCTs), post hoc analyses, and observational cohort studies evaluating biologic agents targeting type 2 pathways or epithelial alarmins in human subjects. The literature focusing primarily on younger asthma cohorts without fixed airflow obstruction was excluded. Data regarding mean chronological age, upper age limits, comorbidity exclusion protocols, and geriatric parameters were systematically extracted and analyzed.

## 2. Pathophysiological Rationale for Biologic Therapies in COPD

Type 2 inflammation is increasingly recognized as a relevant inflammatory endotype in a substantial subset of patients with COPD, being detectable in approximately 20–40% of cases. This endotype is characterized by enhanced expression of IL-4, IL-5, and IL-13, increased eosinophilic infiltration in both blood and airways, and activation of type 2 innate lymphoid cells (ILC2) and Th2 lymphocytes [6]. Unlike the classical neutrophilic inflammation traditionally associated with COPD, type 2 inflammation represents a biologically distinct and potentially treatable mechanism, providing the rationale for the development and implementation of targeted biologic therapies.

### 2.1. Beyond Inhaled Medications: Precision Medicine and Biologic Therapies Targeting the IL-33/TSLP/Type 2 Axis in COPD

Patients with eosinophilic COPD exhibit distinct molecular and transcriptomic profiles. Airway eosinophilia, commonly defined by bronchoalveolar lavage eosinophils > 1%, has been associated with increased epithelial expression of type 2 inflammatory gene signatures, enhanced IL-13 signaling, and markers of mast cell activation [7]. These molecular changes are accompanied by upregulation of pathways typically associated with asthma-related inflammation and are clinically linked to more severe airflow obstruction and more extensive radiographic emphysema compared with non-eosinophilic COPD [8]. Interestingly, inhaled corticosteroid (ICS) treatment administered for 12 weeks has been shown to reduce epithelial expression of type 2 and mast-cell-related inflammatory signatures specifically in eosinophilic patients, supporting the biological responsiveness of this phenotype to anti-inflammatory therapy [8].

At the tissue level, elevated blood eosinophil counts correlate with increased eosinophilic infiltration in sputum, bronchoalveolar lavage fluid, and bronchial mucosal tissue, together with higher concentrations of IL-5 and CCL24 (eotaxin-2). Furthermore, gene expression analyses demonstrated differential expression of several type 2-related markers, including CLCA1, CCL26, IL-13, and CST1, in both sputum cells and bronchial epithelial brushings from patients with higher eosinophil counts. Nevertheless, compared with asthma, the type 2 inflammatory signature observed in COPD appears more restricted and biologically heterogeneous [9].

### 2.2. Eosinophilic COPD and Asthma–COPD Overlap

Eosinophilic COPD is generally identified by elevated blood eosinophil levels, commonly ≥300 cells/μL, together with increased expression of type 2 biomarkers and enhanced responsiveness to corticosteroid therapy [10,11]. Clinically, this phenotype has been associated with accelerated lung function decline, increased exacerbation risk, and distinct airway microbiome characteristics, including reduced predominance of Haemophilus species [9,10].

Asthma–COPD overlap (ACO) represents another clinically important condition characterized by persistent airflow limitation accompanied by features of both asthma and COPD [12,13,14]. Rather than being considered a single disease entity, ACO is now viewed as a heterogeneous syndrome encompassing multiple phenotypes with overlapping inflammatory and clinical characteristics. Epidemiological studies estimate a prevalence ranging from 0.9% to 11.1% in the general population, affecting approximately 15–45% of patients with obstructive airway diseases [12,15]. Compared with patients affected by asthma or COPD alone, individuals with ACO typically exhibit greater symptom burden, worse quality of life, and more frequent and severe exacerbations [12]. Functional assessment often reveals partially reversible airflow obstruction superimposed on persistent baseline obstruction, reflecting the coexistence of asthma-like and COPD-like pathophysiological features [15]. Importantly, treatment strategies in ACO should primarily follow asthma-oriented recommendations because LABA/LAMA therapy without ICS may increase the risk of severe exacerbations and mortality [13,15].

### 2.3. Roles of Key Cytokines and Inflammatory Pathways

IL-4 and IL-13 play a pivotal role in type 2 airway inflammation through signaling mediated by the shared IL-4 receptor α subunit [16,17]. These cytokines contribute to increased fractional exhaled nitric oxide (FeNO) production via inducible nitric oxide synthase activation and promote bronchial hyperreactivity, mucus production, vascular permeability, and tissue injury. They also stimulate pulmonary and activation-regulated chemokine (PARC) production, which has been associated with emphysema severity, systemic inflammation, and cardiovascular complications. Additionally, IL-4 and IL-13 contribute to airway remodeling by promoting fibrosis and smooth muscle alterations while simultaneously driving mucus plug formation, a feature strongly associated with mortality in COPD [16].

IL-5 specifically regulates eosinophil biology by controlling eosinophil differentiation, activation, migration, and survival within blood and tissues [17]. Although eosinophils may contribute to host defense mechanisms against parasites and possibly viral or bacterial pathogens, eosinophil-derived granule proteins such as major basic protein, eosinophil cationic protein, and eosinophil peroxidase may induce epithelial damage, tissue remodeling, and mucus plugging, thereby contributing to disease progression [9].

TSLP and IL-33 act as upstream epithelial alarmins released in response to environmental insults, including allergens, viral infections, helminths, and cigarette smoke-induced epithelial injury [18,19]. These molecules activate ILC2 cells and shape downstream adaptive immune responses, thereby amplifying type 2 inflammation. Ongoing clinical trials are currently evaluating biologic therapies targeting these pathways, including anti-TSLP (tezepelumab), anti-IL-33 (itepekimab and tozorakimab), and anti-ST2 (astegolimab) agents [18,19].

### 2.4. Biomarkers for Patient Stratification

Blood eosinophil count currently represents the most validated and clinically applicable biomarker for identifying type 2 inflammation in COPD and predicting therapeutic responsiveness [9,11,20]. Thresholds of ≥300 cells/μL are generally considered the most reliable for biologic therapy selection, whereas levels of ≥100 cells/μL to 150 cells/μL are associated with increased responsiveness to ICS therapy [20,21]. Blood eosinophils demonstrate acceptable repeatability at lower thresholds, although variability increases at higher values [20]. Elevated eosinophil counts predict greater reductions in exacerbation risk with ICS treatment and are associated with accelerated lung function decline in untreated patients [20,21]. However, blood eosinophils should not be interpreted as a direct surrogate for airway eosinophilia alone but rather as a broader marker of type 2 inflammatory activation, given the imperfect concordance between circulating eosinophils and airway or tissue biomarkers [9,20].

Additional biomarkers involved in type 2 inflammation include elevated FeNO levels, total serum IgE, plasma eotaxin-3, and serum PARC concentrations [16]. Increased FeNO levels (≥20 ppb) reflect activation of the IL-4/IL-13 axis and appear predictive of an enhanced response to dupilumab therapy. Similarly, reductions in serum IgE and eotaxin-3 have been observed following IL-13 pathway blockade [16].

Interestingly, inflammatory phenotypes in COPD also appear associated with distinct airway microbiome patterns. Eosinophilic inflammation is generally linked to more diverse microbial communities without dominant bacterial genera, whereas neutrophilic inflammation is more commonly associated with Haemophilus-dominant dysbiosis [9].

### 2.5. Evidence Supporting Biologic Therapies in COPD

Several biologic agents targeting T2 inflammatory pathways have been investigated in COPD. Clinical trials have demonstrated heterogeneous efficacy across biologic classes, with the most robust evidence currently available for dupilumab and more modest effects observed with anti-IL-5 strategies. Detailed evidence from phase II and phase III studies is discussed in the following section.

### 2.6. Non-Type 2 Inflammation in COPD

Despite the increasing recognition of type 2 inflammation, neutrophilic inflammation remains the predominant inflammatory pattern in COPD and is primarily driven by T1 and T17 immune responses, inflammasome activation, and bacterial colonization [6,22]. Therapeutic strategies targeting non-type 2 pathways include phosphodiesterase inhibitors such as roflumilast and ensifentrine, as well as investigational JAK and MAPK inhibitors [23,24]. However, biologic therapies targeting neutrophilic inflammation have generally produced disappointing results, with some studies even reporting an increased infection risk [22,24]. These findings suggest that neutrophilic inflammation in COPD may often represent a consequence of chronic bacterial colonization and dysbiosis rather than an easily modifiable inflammatory target.

### 2.7. Clinical Implications

Current evidence suggests that biologic therapy may be most beneficial in patients with eosinophilic COPD who continue to experience exacerbations despite optimized inhaled treatment. Patient selection should integrate blood eosinophil counts, exacerbation history, and additional biomarkers of T2 inflammation whenever available.

## 3. Currently Available and Emerging Biologics for COPD

**Figure 1:**
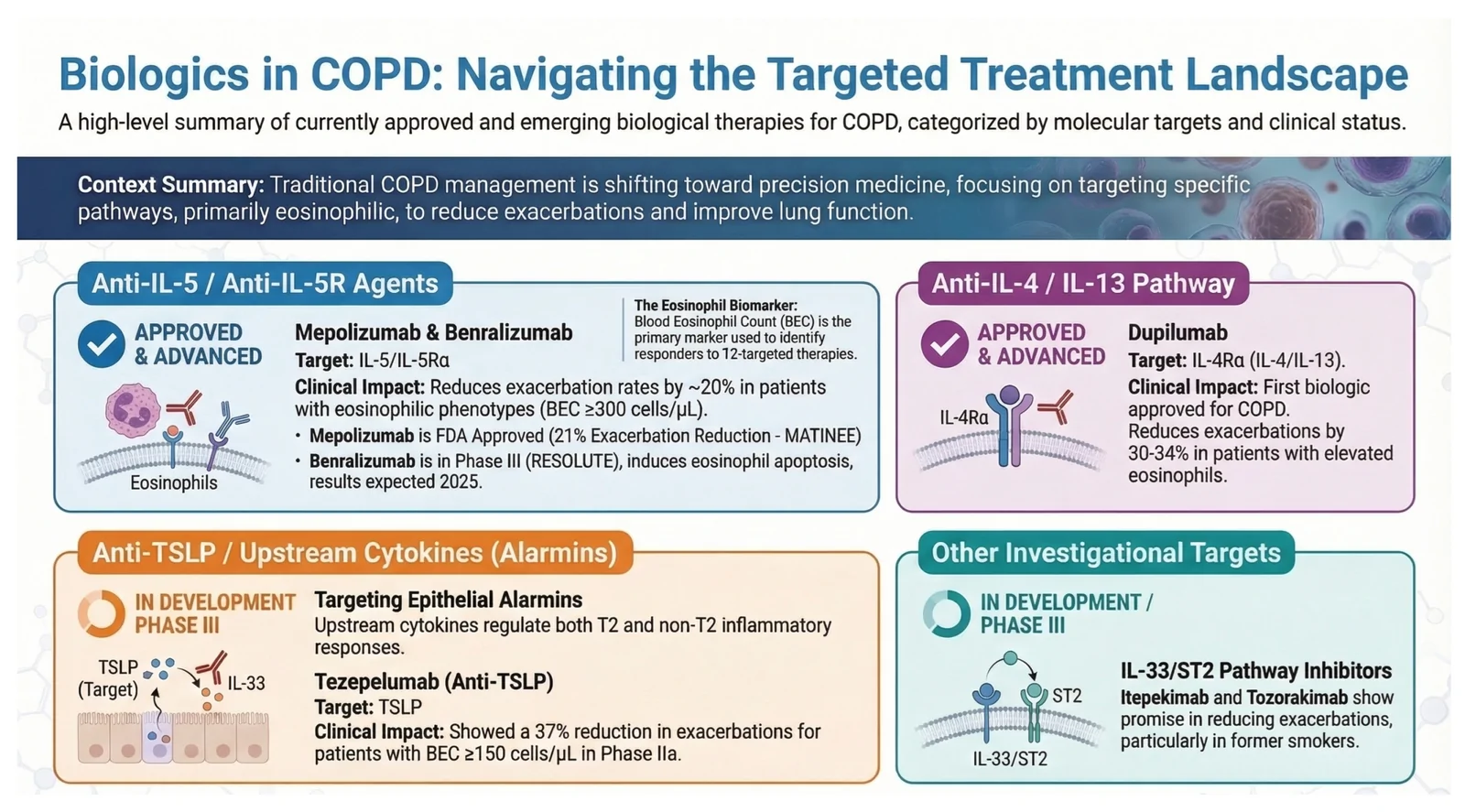
Schematic representation of currently available and emerging biologic therapies for COPD.

Based on the pathophysiological rationale discussed above, several biologics have been investigated in eosinophilic COPD (see Figure 1).

### 3.1. Anti-IL-5 and Anti-IL-5R Agents

#### 3.1.1. Mepolizumab

A humanized monoclonal IgG1 antibody that binds directly to circulating IL-5, preventing its interaction with the IL-5 receptor alpha (IL-5Rα) on eosinophils [24,25]. Early phase 3 trials, including METREX and METREO, demonstrated a modest 14% to 20% reduction in the annual rate of moderate-to-severe exacerbations in COPD patients with an eosinophilic phenotype [18,26]. More recently, the comprehensive phase 3 MATINEE trial established that mepolizumab significantly reduced the annualized rate of moderate or severe exacerbations by 21% in patients with blood eosinophil counts of ≥300 cells/μL, leading to its approval by the US Food and Drug Administration (FDA) as an add-on maintenance treatment for inadequately controlled COPD with an eosinophilic phenotype [24,27].

#### 3.1.2. Benralizumab

A humanized, afucosylated mAb that binds directly to the IL-5Rα subunit, uniquely depleting eosinophils rapidly through antibody-dependent cell-mediated cytotoxicity (ADCC) [28,29]. The initial phase 3 GALATHEA and TERRANOVA trials failed to demonstrate a statistically significant reduction in overall COPD exacerbation rates [18,30]. However, subsequent post hoc analyses identified a highly responsive subgroup: patients with baseline blood eosinophils ≥ 220 cells/μL to 300 cells/μL, a history of three or more previous-year exacerbations, and baseline triple therapy experienced a significant reduction in exacerbation risk [24,28]. Furthermore, the phase 2 ABRA trial demonstrated benralizumab’s acute efficacy, showing a four-fold reduction in treatment failure when administered during acute eosinophilic exacerbations of COPD compared to standard systemic glucocorticoids [24,31].

### 3.2. Anti-IL-4/IL-13 Pathway Inhibitors

#### 3.2.1. Dupilumab

A fully human monoclonal antibody that binds to the IL-4Rα subunit, thereby inducing dual blockade of both IL-4 and IL-13 downstream signaling via the JAK-STAT pathway [32,33]. Dupilumab is the first biologic to receive comprehensive regulatory approval (FDA and EMA) for COPD [24,34]. Its approval was based on two landmark phase 3 trials, BOREAS and NOTUS [17,33]. Both trials enrolled patients on triple therapy with symptomatic chronic bronchitis and a blood eosinophil count of ≥300 cells/μL [35]. Dupilumab treatment resulted in a remarkable 30% to 34% reduction in the annualized rate of moderate-to-severe exacerbations [17,33]. Additionally, it demonstrated rapid, clinically meaningful improvements in pre-bronchodilator FEV1 (increasing by approximately 160 mL) and significantly ameliorated health-related quality of life (SGRQ scores) [17,33]. Pathologically, its efficacy is linked to reversing mucus plugging and dynamic lung hyperinflation [32,36].

#### 3.2.2. Lebrikizumab and Tralokinumab

Both of these agents are monoclonal antibodies selectively targeting IL-13 [37]. Clinical trials exploring lebrikizumab in COPD patients with frequent exacerbations failed to show a significant reduction in exacerbation rates compared to placebo, suggesting that isolated IL-13 blockade may be insufficient compared to the dual-pathway inhibition achieved by dupilumab [24].

### 3.3. Anti-TSLP and Upstream Cytokine Inhibitors (Anti-Alarmins)

#### 3.3.1. Tezepelumab (Anti-TSLP)

Evaluated in the phase 2a COURSE trial in moderate-to-very-severe COPD patients [38]. While the trial did not meet its primary endpoint in the overall population (showing a non-significant 17% reduction in exacerbations), tezepelumab demonstrated profound efficacy in biomarker-enriched subgroups, yielding a 37% reduction in exacerbations for patients with eosinophils ≥150 cells/μL and a 46% reduction in those with ≥300 cells/μL [38].

#### 3.3.2. Anti-IL-33 Agents (Itepekimab and Tozorakimab)

Itepekimab (anti-IL-33 mAb) demonstrated phase 2a efficacy predominantly in former smokers, substantially reducing exacerbations and improving lung function, leading to the ongoing phase 3 AERIFY trials [30,39]. Tozorakimab exhibits dual pharmacology by inhibiting both the ST2-dependent signaling of reduced IL-33 and the RAGE/EGFR signaling of oxidized IL-33, thereby addressing both systemic inflammation and epithelial dysfunction [40,41]. The phase 2a FRONTIER-4 trial demonstrated positive efficacy signals in FEV1 improvement and composite exacerbation reduction specifically in a subgroup with ≥2 prior exacerbations, paving the way for the phase 3 TITANIA and OBERON trials [37,40].

#### 3.3.3. Astegolimab (Anti-ST2 Receptor)

Blocks the IL-33 receptor [30]. In the COPD-ST2OP phase 2a trial, it did not significantly reduce the overall exacerbation rate but significantly improved health status and quality of life [24,30].

### 3.4. Other Investigational Targets

Early biological research in COPD primarily targeted non-T2, type 1, and type 3 neutrophilic inflammatory pathways, but these broad immunosuppressive strategies largely failed to demonstrate clinical efficacy [30].

#### 3.4.1. Anti-TNF-α (Infliximab), Anti-IL-1β/IL-1R1 (Canakinumab, MEDI8968), Anti-IL-17A (CNTO6785), and Anti-IL-8 (ABX-IL8)

Trials evaluating these agents in unselected COPD populations uniformly failed to reduce exacerbations or improve lung function [29,30]. In some instances, such as with Infliximab, therapy was associated with an unacceptable increase in malignancies and pneumonia [30,42].

#### 3.4.2. Small Molecule Inhibitors

Beyond monoclonal antibodies, small molecules targeting intracellular signaling are emerging [24]. Ensifentrine, a first-in-class dual PDE3/PDE4 inhibitor, has recently received FDA approval as it effectively combines bronchodilation with broad anti-inflammatory effects, reducing exacerbations while avoiding the severe gastrointestinal side effects of oral PDE4 inhibitors like roflumilast [24,43]. Inhibitors of the p38 MAPK and PI3Kδ pathways (e.g., acumapimod, nemiralisib) remain in various developmental stages but have historically suffered from unfavorable benefit-to-risk ratios.

### 3.5. Comparison with Biologic Use in Severe Asthma

While the advent of T2-targeted biologics has radically transformed the therapeutic landscape of severe asthma, bridging these successes to COPD has proven highly complex due to distinct immunopathological differences [30,44].

In severe eosinophilic asthma, anti-IL-5/IL-5R agents (mepolizumab, benralizumab) yield dramatic exacerbation reductions (often ≥50%) and oral corticosteroid-sparing effects [28,45]. Conversely, the magnitude of benefit for these same agents in COPD is considerably blunted (reductions of 15–20%), even when matched for equivalent blood eosinophil concentrations [26,30].

The constrained efficacy of narrow IL-5 blockade in COPD implies that eosinophilia in COPD does not act in isolation [41]. Instead, COPD is characterized by highly redundant inflammatory loops where neutrophilic inflammation, macrophage activation, oxidative stress, and chronic structural remodeling (emphysema) dominate [18,41].

Due to this inflammatory redundancy, biologics that block multiple pathways concurrently—such as dupilumab (blocking both IL-4 and IL-13) or tezepelumab (blocking the master regulator TSLP)—appear more effective in COPD than selectively targeting eosinophil survival via IL-5 [18,41]. Furthermore, whereas biologic therapy in asthma is often deployed across diverse age groups to achieve disease remission, biologic application in COPD is currently confined to a heavily pre-treated, older demographic with irreversible structural lung damage, highlighting an urgent need for early biomarker-directed intervention to alter the disease trajectory [30,46].

## 4. Evidence from Phase III Clinical Trials

Evidence emerging from randomized clinical trials (RCTs) has reshaped the follow-up pharmacological treatment of COPD. Indeed, Global Initiative for Chronic Obstructive Lung Disease has proposed the introduction of dupilumab in 2025, and mepolizumab in 2026, as recommended add-on therapies for COPD patients with a high blood eosinophil count (BEC) experiencing moderate or severe exacerbations despite receiving triple inhaler therapy.

The first published phase III RCTs have been the Mepolizumab vs. Placebo as Add-on Treatment for Frequently Exacerbating COPD Patients (METREX) and the Mepolizumab vs. Placebo as Add-on Treatment for Frequently Exacerbating COPD Patients Characterized by Eosinophil Level (METREO) [26]. Both trials enrolled patients with a diagnosis of COPD, according to the American Thoracic Society-European Respiratory Society recommendation, with an FEV1 between 20 and 80% and a history of ≥2 moderate exacerbation or ≥1 severe exacerbation with hospitalization in the previous year; patients ought to receive inhaled corticosteroids plus two additional medications for the twelve months prior to screening and triple inhaler therapy for a minimum of three months. Those with a current diagnosis of asthma and non-smokers with a history of asthma were excluded. METREO enrolled only patients with a BEC ≥ 150 cells/μL at screening and a BEC ≥ 300 cells/μL in the previous year, while METREX did not consider BEC as an inclusion criterion, although patients were stratified after randomization according to their BEC level. METREX randomized patients with a 1:1 ratio to mepolizumab 100 mg and placebo, while METREO with a 1:1:1 ratio to mepolizumab 300 mg, mepolizumab 100 mg and placebo. Overall, the primary endpoint (i.e., mean annual rate of moderate or severe exacerbations) and the secondary endpoints, including the St. George’s Respiratory Questionnaire (SGRQ) total score and the COPD Assessment Test (CAT) score, were not met in both trials. Nevertheless, a significant reduction in the mean annual rate of moderate or severe exacerbations was observed in the modified intention-to-treat population with an eosinophilic phenotype in METREX (1.40/year versus 1.71/year; RR 0.82, 95% CI 0.68–0.98; adjusted *p* = 0.04).

The evidence that eosinophilic COPD patients are more likely to benefit from mepolizumab prompted the authors of the Mepolizumab as Add-on Treatment in Participants with COPD Characterized by Frequent Exacerbations and Eosinophil Level (MATINEE) to include only patients with a BEC ≥ 300 cells/μL at screening and a BEC ≥ 150 cells/μL in the previous year. Patients were randomized with a 1:1 ratio to mepolizumab 100 mg and placebo and the treatment arm showed a significant reduction in the primary endpoint of annualized rate of moderate or severe exacerbations (0.80/year versus 1.01/year; RR 0.79, 95% CI 0.66–0.94; adjusted *p* = 0.01), in the annualized rate of exacerbations resulting in emergency department visit and/or hospitalization (0.13/year versus 0.20/year; RR 0.65, 95% CI 0.43–0.96), and in the time to first moderate or severe exacerbation (hazard ratio 0.77, 95% CI 0.64–0.93), but not in the SGRQ total score and CAT score. Overall, the rate of exacerbations was lower due to the COVID-19 pandemic that led to an extension of the follow-up [27].

In contrast to mepolizumab, the anti-IL5R benralizumab did not demonstrate clinical efficacy in COPD patients. The Benralizumab Efficacy in Moderate to Very Severe Chronic Obstructive Pulmonary Disease with Exacerbation History (GALATHEA) and the Efficacy and Safety of Benralizumab in Moderate to Very Severe Chronic Obstructive Pulmonary Disease with Exacerbation History (TERRANOVA) enrolled COPD patients with a history of ≥2 moderate exacerbation or ≥1 severe exacerbation with hospitalization in the previous year receiving dual or triple therapy; patients with a diagnosis of asthma were excluded only if asthma was judged to contribute to respiratory symptoms. BEC was not considered an inclusion criterion, although patients were stratified in three groups using 220 cells/μL and 300 cells/μL as cut-offs. In both trials the annual rates of COPD exacerbations associated with benralizumab given as add-on maintenance treatment did not differ among groups, as well as the change from baseline in prebronchodilator FEV1 and the SGRQ [47].

Consistent with the MATINEE and the preceding METREX and METREO trials, the RESOLUTE (NCT04053634) has restricted enrollment to patients with a blood eosinophil count 300 cells/μL at screening and a documented historical count 150 cells/μL within the prior 52 weeks (or confirmed during the run-in period). The trial ended in 2025, but the results have not yet been published.

Dupilumab was the first biologic therapy approved for COPD, based on the results of the Dupilumab for COPD with Type 2 Inflammation Indicated by Eosinophil Counts (BOREAS) and Dupilumab for COPD with Blood Eosinophil Evidence of Type 2 Inflammation (NOTUS) phase III trials. In BOREAS trial, symptomatic COPD patients with an FEV1 between 30 and 70%, a history of ≥2 moderate exacerbation or ≥1 severe exacerbation with hospitalization in the previous year and a BEC 300 cells/μL at the screening were randomized with a 1:1 ratio to dupilumab 300 mg and placebo. Patients were required to receive triple inhaler therapy for at least three months before screening and a current or history diagnosis of asthma was an exclusion criterion. In contrast with RCTs of mepolizumab, patients had to have had signs or symptoms of chronic bronchitis for at least 3 months during the year before screening and enrollment of current smokers were capped at 30%. The primary endpoint of annualized rate of moderate or severe exacerbations of COPD was met, with an adjusted rate of 0.78 (95% CI 0.64–0.93) in the dupilumab group and 1.10 (95% CI 0.93–1.30) in the placebo group and a rate ratio of 0.70 (95% CI 0.58–0.86; *p* < 0.001), as well as the secondary outcomes (i.e., change in prebronchodilator FEV1, SGRQ total score and E-RS-COPD total score) [17].

These positive results were confirmed in the NOTUS trial, in which COPD patients, in addition to the BOREAS inclusion and exclusion criteria, were required to have had at least one exacerbation while receiving background triple inhaler therapy. The annualized rate of moderate or severe exacerbations of COPD was lower in the dupilumab group than in the placebo group (rate ratio 0.66, 95% CI 0.54–0.82; *p* < 0.001), and the SGRQ total score was decreased in the treatment arm; in contrast, there was no significant difference in the E-RS-COPD total score between the groups [17].

To date, there are no published phase III RCTs on the efficacy and safety of monoclonal antibodies targeting alarmins. The anti-thymic stromal lymphopoietin (TSLP) tezepelumab failed to significantly reduce the annualized rate of moderate or severe COPD exacerbations in the phase IIa RCT (COURSE) [38], so the phase III EMBARK (NCT06883305) and JOURNEY (NCT06878261) RCTs on tezepelumab were designed to include only eosinophilic COPD patients, using ≥150 cells/μL as a cut-off. Studies are ongoing, and completion is estimated in 2029. In contrast, in the upcoming months the results of the phase III RCTs on anti-IL33 itepekimab and tozorakimab will be available. Both biologics failed to meet their primary outcome in the phase IIa RCTs, respectively the annualised rate of moderate-to-severe acute exacerbations of COPD during the treatment period and the improvement in the pre-bronchodilator FEV1 from baseline to week 12 [40,48]. Notably, itepekimab reduced exacerbation rate (rate ratio 0.58, 95% CI 0.39–0.85) and improved lung function in former smokers with COPD, while tozorakimab showed positive efficacy in the subgroup of patients with COPD with a high risk of exacerbations hazard ratio (0.61, 80% CI 0.37–1.00). This explains why the AERIFY-1/2 (NCT04701983/NCT04751487) on itepekimab restricted the enrollment to COPD patients with smoking cessation occurred ≥6 months prior to screening (smokers are allowed only in AERIFY-2), while the enrollment of COPD patients in the OBERON (NCT05166889) and TITANIA (NCT05158387) phase III RCTs focused on COPD frequent exacerbators. All these studies on anti-alarmin did not consider BEC as an inclusion criterion.

Finally, in 2027 are expected the results of the ARNASA (NCT05595642) phase III RCT on the anti-ST2 astegolimab, which demonstrated in the phase IIb ALIENTO trial a significant reduction in the annualized rate of moderate/severe COPD exacerbations astegolimab 476 mg Q2W group [49].

In conclusion, no safety signals have so far emerged from randomized controlled trials on biologic therapies.

## 5. Biologics in the Elderly COPD Population: An Underexplored Area

### 5.1. Epidemiology of COPD in Older Adults

Age is a key determinant in the epidemiology of COPD, with prevalence increasing progressively across the lifespan. Population-based analyses demonstrate a marked age-related gradient in disease burden. Global estimates indicate that COPD prevalence rises from 2.8% among individuals aged 30–34 years to 4.95% in those aged 35–39 years, increasing sharply in later decades of life to 15.8% in individuals aged 60–64 years, 20.3% in those aged 65–69 years, 23.4% in the 70–74-year age group, and reaching 27.8% among adults aged 75–79 years [50]. Consistent with this pattern, the greatest absolute number of COPD cases worldwide occurs among individuals aged 60–69 years across multiple regions, including the Americas, Europe, South-East Asia, and the Western Pacific [50]. Similar age-related trends have been reported in national data from the United States, where COPD prevalence increases from 0.4% among adults aged 18–24 years to 10.5% among individuals aged 75 years and older [51]. The variance between estimated global prevalence (27.8%) and reported national prevalence (10.5%) may reflect differences in diagnostic methodology, such as spirometric screening versus clinical diagnosis or self-reporting. These findings underscore the strong association between advancing age and COPD burden, highlighting older adults as a population disproportionately affected by the disease.

### 5.2. Immunosenescence and Inflammaging

Aging exerts profound effects on immune function and is a central factor in the pathobiology of chronic inflammatory diseases such as COPD. The aging immune system undergoes a process of immune remodeling characterized by two interrelated phenomena: immunosenescence and inflammaging. Immunosenescence refers to the progressive decline in both innate and adaptive immune responses, whereas inflammaging describes the concomitant state of chronic, low-grade systemic inflammation [52,53]. Together, these processes reflect a shift from effective host defense toward dysregulated and persistent inflammatory signaling. At the cellular level, immunosenescence is characterized by substantial alterations in T-cell compartments, including reduced thymic output, contraction of the naïve T-cell pool, and expansion of oligoclonal memory T cells [54,55]. These changes are particularly pronounced in CD8+ T cells, which exhibit increased loss of the co-stimulatory molecule CD28, resulting in the accumulation of highly differentiated, pro-inflammatory CD28-null populations. In individuals aged ≥ 60 years, CD28-null cells may account for up to 53% of CD8+ T cells [56]. Consistent with this aging phenotype, patients with COPD demonstrate an increased proportion of memory T-cell subsets, particularly CD8+ central memory cells, suggesting an overlap between disease-related and age-related immune remodeling [57,58]. These shifts contribute to impaired pathogen clearance alongside enhanced pro-inflammatory activity. Inflammaging further amplifies this dysregulated immune state. It is characterized by elevated circulating levels of pro-inflammatory mediators, including interleukin IL-1, IL-6, and TNF-α, driven in part by cumulative lifelong exposure to infectious and non-infectious antigens [59,60]. In COPD, this baseline inflammatory milieu is further intensified by disease-specific mechanisms. Senescent structural and immune cells—including alveolar epithelial cells, endothelial cells, and macrophages—adopt a senescence-associated secretory phenotype, releasing pro-inflammatory cytokines and proteases that perpetuate local and systemic inflammation [61,62]. In particular, increased production of IL-6 and IL-8 by senescent alveolar cells has been linked to sustained inflammatory signaling within lung tissue [63,64]. Elevated systemic IL-6 levels in COPD further support its role as a biomarker of both disease activity and age-related immune dysregulation [65]. Additional age-related alterations, including impaired epithelial barrier function, reduced mucociliary clearance, and decreased production of antimicrobial peptides, further compromise pulmonary defense mechanisms and promote persistent inflammation [66,67,68]. These age-related immune alterations have important implications for biologic therapies in COPD. Immunosenescence may blunt therapeutic responsiveness by limiting immune plasticity and altering cytokine networks, while inflammaging may modify treatment effects by sustaining redundant inflammatory pathways. Consequently, biologic agents targeting specific cytokines (e.g., type 2 inflammation pathways) may exhibit variable efficacy in older patients, depending on the relative contribution of age-related versus disease-specific inflammation. At the same time, by attenuating key inflammatory mediators and reducing exacerbation frequency, biologics may partially counteract the systemic inflammatory burden associated with inflammaging. Overall, the interplay between immunosenescence, inflammaging, and COPD underscores the need for age-specific therapeutic strategies. A deeper understanding of these mechanisms may improve patient selection, optimize biologic treatment responses, and support the development of precision medicine approaches tailored to the older COPD population.

### 5.3. Frailty in Older Adults with COPD

Frailty is highly prevalent in older adults with COPD, affecting approximately 32% of patients (with an additional 56% being prefrail), and is associated with substantially worse clinical outcomes including accelerated disease progression, increased exacerbations, higher mortality, and reduced life expectancy [69,70]. The 2026 GOLD guidelines now recognize frailty as an important consideration in COPD management, defining it by five components: weakness, slowness, exhaustion, low physical activity, and unintentional weight loss [20]. Older adults with COPD have a two-fold increased odds of frailty compared to those without COPD (pooled OR 1.97, 95% CI 1.53–2.53). In the Singapore Longitudinal Ageing Study, baseline prevalence of frailty in COPD patients was 6.8% compared to lower rates in non-COPD participants (OR 1.61, 95% CI 1.15–2.26) [70,71].

The 2025 UK Biobank study demonstrated important interactions regarding disease development, highlighting a significant interaction between genetic predisposition and frailty in COPD development (p=0.0252), as well as a significant interaction between preserved ratio impaired spirometry (PRISm) and frailty (p=0.0027). Furthermore, the longitudinal data indicate that frailty and prefrailty significantly increase the risk of incident COPD, yielding hazard ratios of 2.21 (95% CI: 2.06–2.37) for frail individuals and 1.45 (95% CI: 1.39–1.51) for prefrail individuals, respectively.

This suggests a bidirectional relationship where frailty both increases COPD risk and is worsened by COPD [69,72].

Notably, frail COPD patients demonstrate a lower FEV_1_ (mean difference −5.0%, 95% CI −6.70 to −3.42%), reduced 6 min walk distance (mean difference −90.23 m, 95% CI −124.70 to −55.76), poorer activities of daily living (standardized mean difference −0.99, 95% CI −1.35 to −0.62), higher symptom burden with a CAT score increased by 6.2 points (95% CI 4.43–7.96) and mMRC grade by 0.93 (95% CI 0.85–1.02) [70]. In addition, frail COPD patients exhibit significantly lower physical activity levels and worse health-related quality of life (adjusted HR 1.4, 95% CI 0.97–2.0) [70,73].

In the Singapore study, frailty combined with FEV_1_ < 80% or dyspnea predicted a threefold to fourfold increased odds of prevalent and incident instrumental and basic ADL disability and a twofold to threefold increased mortality hazard. Moreover, a summary score combining physical frailty, FEV_1_ < 80%, and dyspnea predicted sevenfold to 8.5-fold increased risks in the highest risk category [71].

The European Respiratory Society (ERS) Statement recommends multidimensional interventions including geriatric care integration, rehabilitation (exercise-based), nutritional support, pharmacological therapies (optimizing COPD and comorbidity management) and psychological therapies (addressing anxiety, depression, andloss of confidence). Furthermore, the 2026 GOLD guidelines recommend evaluating the “3 Ms” in older COPD patients with multimorbidity and frailty: Mentation (cognitive function assessment), Medication (comprehensive medication review) and Mobility (Functional assessment) [20]. This should be followed by systematic evaluation of common comorbidity clusters that independently impact outcomes.

The 2023 ERS statement outlines two primary frameworks for evaluating frailty in clinical and research settings [73]. The first framework, the Physical Frailty Phenotype based on the Fried criteria, remains the most widely adopted approach. This model evaluates five specific domains: wasting (indicated by unintentional weight loss), slowness (measured via gait speed), weakness (assessed through grip strength), low physical activity, and exhaustion. Within this phenotype, frailty is diagnosed by the presence of deficits in three or more domains, whereas prefrailty is defined by deficits in one or two domains. The second framework comprises cumulative deficit models, which generate a Frailty Index. This index computes the ratio of accumulated deficits—including clinical symptoms, laboratory findings, disabilities, and comorbidities—relative to the total number of items assessed. In this model, a threshold score of 0.25 or higher typically denotes frailty.

Beyond these two main frameworks, several other validated instruments are available for patients with chronic obstructive pulmonary disease (COPD). These include the Short Physical Performance Battery (SPPB), a 5 to 10 min test assessing balance, gait speed, and sit-to-stand strength; the Clinical Frailty Scale, a brief, interview-based tool scored from 1 to 7 that requires fewer than 3 min; the Edmonton Frail Scale, a more comprehensive 10 to 15 min evaluation incorporating a timed get-up-and-go test and a clock-drawing task; and the FRAIL scale, a rapid 3 to 5 min self-report screening tool. Additionally, the Hospital Frailty Risk Score leverages administrative data to facilitate population-level risk assessment [74]. Reflecting this growing clinical focus, the 2026 Global Initiative for Chronic Obstructive Lung Disease (GOLD) guidelines explicitly recognize frailty as a critical factor in COPD management, defining the condition through the five core components of weakness, slowness, exhaustion, low physical activity, and unintentional weight loss [20].

### 5.4. Nutritional Status, Physical Therapy and Rehabilitation in Older Adults

Low BMI and particularly low fat-free mass are associated with worse outcomes in COPD patients [20]. Nutritional status is a critical prognostic factor for morbidity and mortality, with involuntary weight loss and decreased fat-free mass associated with increased mortality. COPD patients may display different body composition phenotypes ranging from cachexia to obesity to sarcopenia, making comprehensive assessment of body composition essential to determine muscle mass and identify potential health risks [75]. The 2026 GOLD guidelines and recent evidence state that oral nutritional supplements are the mainstay of nutritional support in stable, malnourished COPD patients [20,76]. A 2021 randomized controlled trial demonstrated that high-protein oral supplements containing β-hydroxy-β-methylbutyrate (HMB) for up to 90 days after hospital discharge resulted in 71% lower mortality compared with controls in stable malnourished older adults with COPD (*p* = 0.0395) [76]. In malnourished COPD patients specifically, nutritional supplementation demonstrates significant improvements in 6 min walk distance (6MWD), enhanced respiratory muscle strength, and improved health status and quality of life [20,75]. For patients unable to meet nutritional needs orally, enteral tube feeding has demonstrated benefits including greater weight gain, improved maximal expiratory pressure, and significantly improved arterial oxygen and carbon dioxide levels compared with minimal oral intake [76].

Current evidence supports a comprehensive strategy combining nutritional support with pulmonary rehabilitation and pharmacological treatments. The combination of nutritional intervention with physical exercise appears particularly effective in improving muscle strength and exercise tolerance [75].

Pulmonary rehabilitation significantly improves multiple measures of exercise capacity: 6 min walk distance (6MWD) with a mean improvement of 43.93 m (95% CI, 32.64–55.21), incremental shuttle walk distance with an improvement of 39.77 m (95% CI, 22.38–57.15) and peak work capacity with an improvement of 6.77 watts (95% CI, 1.89–11.65) [20].

### 5.5. Cognitive Impairment

Cognitive dysfunction represents a highly prevalent and clinically significant comorbidity among older adults with chronic obstructive pulmonary disease (COPD), affecting an estimated 32% of this population on average [77]. When subjected to extensive neuropsychological testing, the detectable rate of cognitive impairment escalates substantially, with evidence suggesting that up to 56% to 60% of patients exhibit measurable deficits [77]. This profound cognitive burden severely impacts daily functioning, attenuates treatment adherence, and adversely modifies overall clinical outcomes [77]. Recent meta-analytic data underscore this vulnerability, demonstrating that individuals with COPD face a 24% increased risk of developing dementia (RR 1.24, 95% CI 1.03–1.50) and a 30% elevated risk of cognitive impairment (RR 1.30, 95% CI 1.13–1.49) compared to age-matched controls, culminating in a 1.74-fold higher risk of generalized cognitive decline [77]. While no significant sex-based differences in dementia risk have been identified within this population, the incidence demonstrates a strong positive correlation with advancing age [77].

At the domain-specific level, COPD-related cognitive impairment does not manifest uniformly but predominantly affects distinct neuropsychological axes [78]. Key vulnerabilities are observed in executive function, disrupting critical processes such as planning, strategic decision-making, and cognitive flexibility [78]. Furthermore, patients frequently demonstrate impaired attention, characterized by deficits in sustained concentration and focus, alongside compromised memory pathways that affect working memory capacity and information recall [78]. Visuospatial abilities, including spatial processing and visual perception, are similarly degraded, altogether contributing to multifaceted cognitive decline [78].

The functional consequences of this cognitive dysfunction are substantial and pervasive, precipitating a marked decline in basic activities of daily living (ADLs) and instrumental ADLs, such as financial management and complex medication scheduling. This loss of functional independence shifts patients toward a state of heightened disability and care dependence. Crucially, cognitive impairment initiates a vicious cycle that directly undermines therapeutic adherence. Patients with cognitive deficits exhibit a significantly higher propensity for critical inhaler technique errors and display poor overall adherence to complex therapeutic plans. This insufficient adherence is further compounded by a reduced capacity for self-management, which severely impairs the patient’s ability to recognize and appropriately respond to acute changes in respiratory symptoms in a timely manner [20,77].

Consequently, the intersection of cognitive decline and COPD drives disproportionate healthcare utilization. The coexistence of these conditions is strongly associated with an elevated risk of hospitalization, prolonged lengths of stay during admissions for acute exacerbations, and higher all-cause readmission rates [20,77]. Ultimately, this systemic vulnerability translates into a severely compromised health-related quality of life, a greater overall disease burden, and a significantly increased risk of mortality, establishing poor cognitive performance as a critical determinant of death in older adults with COPD [20,77].

### 5.6. Comorbidities, Multimorbidity, and the Treatable Traits Framework

Chronic obstructive pulmonary disease in older adults is characterized by a high burden of multimorbidity, which has important implications for the effectiveness of emerging biologic therapies. Older patients commonly present with coexisting metabolic, cardiovascular, psychiatric, and respiratory conditions, all of which influence disease expression, increase exacerbation risk, and may alter treatment responsiveness [79]. As a result, most patients considered for biologic therapy in real-world settings represent a clinically complex population with overlapping systemic conditions. This complexity is further amplified by age-related polypharmacy, which is highly prevalent in older patients with COPD and poses a significant challenge for the implementation of biologic treatments. The use of multiple medications is associated with reduced adherence, increased risk of drug–drug interactions, and cumulative adverse effects, all of which may compromise therapeutic effectiveness [80,81]. In addition, physical frailty and cognitive impairment may further reduce adherence to both inhaled therapies and biologic agents. Concerns about adverse effects, socioeconomic barriers, and limited access to care also contribute to suboptimal treatment persistence, potentially attenuating the real-world benefits of biologic interventions [82].

Among comorbidities, cardiovascular disease is particularly relevant when considering biologic therapy in older patients with COPD. COPD is an established risk factor for cardiovascular morbidity and mortality [83], and the coexistence of these conditions is associated with worse functional status and higher hospitalization rates. Importantly, acute exacerbations—one of the primary targets of biologic therapy—are strongly linked to increased cardiovascular risk, including myocardial infarction and cardiac dysfunction [84]. Patients with frequent or severe exacerbations, who are the main candidates for biologics, are therefore also those at greatest risk of adverse cardiovascular outcomes. In this context, biologic therapies may provide benefits that extend beyond exacerbation reduction. For example, dupilumab, through inhibition of IL-4/IL-13 signaling, reduces airway inflammation, mucus hypersecretion, and exacerbation frequency, which may theoretically contribute to a secondary reduction in hypoxia and systemic inflammation. These effects could translate into a lower risk of cardiovascular complications, including heart failure, particularly in high-risk older populations [85,86]. The observed reduction in adverse events with dupilumab further supports its potential to mitigate downstream cardiovascular risk. However, robust evidence in older populations with multimorbidity remains limited. These considerations highlight the importance of a comprehensive, individualized assessment prior to initiating biologic therapy in COPD. Increasingly, COPD is recognized as a systemic disease in which multimorbidity clusters, rather than spirometric severity alone, drive prognosis, healthcare utilization, and mortality [87]. Accordingly, integrated care strategies that address comorbidities, medication burden, and adherence are essential to optimize biologic treatment outcomes in older patients. The treatable traits approach provides a useful framework for this purpose, supporting a shift toward precision medicine in COPD management. By identifying and prioritizing modifiable pulmonary, extrapulmonary, and behavioral traits, this approach enables more targeted use of biologic therapies and facilitates ongoing reassessment as patient characteristics evolve [88]. Biomarkers—particularly blood eosinophil count, fractional exhaled nitric oxide (FeNO), and emerging multi-omic signatures—are central to this strategy, allowing improved patient selection, prediction of therapeutic response, and monitoring of treatment effectiveness [89,90].

### 5.7. Evidence from Clinical Trials

The efficacy of biologic therapies in COPD has been established primarily through phase II and phase III randomized clinical trials, which are discussed in detail in the previous sections. However, interpretation of these data in older adults requires caution because older individuals remain substantially underrepresented in pivotal studies (see Table 1). Therefore, the major question is no longer whether biologics are effective in selected eosinophilic COPD populations but rather how these results can be translated to real-world older patients characterized by multimorbidity, polypharmacy, frailty, and age-related immune dysfunction.

### 5.8. Representation of Older Adults in Landmark Biologic Trials: A Critical Evidence Gap

Despite the disproportionate burden of COPD in older adults—with prevalence rising to nearly 28% in the 75–79-age group—the landmark randomized controlled trials that form the evidence base for current biologic therapies have systematically underrepresented this population. This discrepancy constitutes a fundamental challenge to the external validity of existing clinical guidelines when applied to real-world older patients. In both the BOREAS and NOTUS phase 3 trials of dupilumab, the upper age limit for enrollment was set at 85 years, and the mean age of enrolled participants was approximately 65 years—a full decade younger than the average age of COPD patients most likely to require add-on biologic therapy in clinical practice [50,51]. A nearly identical demographic profile was observed in the METREX and METREO trials for mepolizumab, which enrolled 1510 patients with a mean age of 65 years (SD 9), further confirming the systematic exclusion of the oldest-old population from pivotal biologic research [26].

Moreover, patients with significant multimorbidity—including poorly controlled cardiovascular disease, active malignancy, immunodeficiency, and certain pulmonary comorbidities such as bronchiectasis—were explicitly excluded from participation. Patients with cognitive impairment, physical frailty, and significant polypharmacy—characteristics that typify older COPD patients—were not systematically assessed or stratified. As a result, age-specific subgroup analyses in these trials, where reported, are based on small numbers of patients aged ≥ 75 years and are generally underpowered to detect differential treatment effects in the oldest segment of the COPD population. This pattern of underrepresentation is not unique to COPD biologics but reflects a broader and longstanding structural limitation of pharmaceutical clinical trial design. A systematic review of 4341 randomized controlled trials published between 1998 and 2015 in the highest-impact medical journals found that 29% of trials specified upper age limits, 92.8% of which provided no scientific justification for this criterion; over the entire 18-year observation period, only a modest and statistically limited reduction in the proportion of such unexplained age exclusions was observed [91]. Similarly, a 2024 analysis of the current status of older adult inclusion in drug development concluded that, despite regulatory mandates from the FDA and EMA encouraging the enrollment of patients representative of the intended treatment population, older adults—particularly those aged ≥75 years—remain significantly underrepresented in pivotal trials across therapeutic areas. Key barriers identified include the systematic exclusion of multimorbidity, the use of polypharmacy as an exclusion surrogate, stringent performance status criteria, and the logistical complexity of enrolling frail or cognitively impaired patients [92]. In the specific context of COPD pharmacological trials, a recent systematic review and meta-analysis of 190 randomized controlled trials published between 2010 and 2024 confirmed that women comprised only 31.7% of enrolled participants (pooled enrollment disparity difference −0.21), with age identified as a significant moderator of enrollment inequality: trials enrolling older patients exhibited proportionally greater demographic underrepresentation [93]. While this analysis focused on sex-based disparities, the concurrent finding that age amplifies the gap between trial populations and disease burden populations is directly applicable to the broader problem of geriatric underrepresentation in COPD research. The clinical consequences of this evidence gap are substantial. Current biologic treatment guidelines—including the 2026 GOLD (Global Initiative for Chronic Obstructive Lung Disease) recommendations endorsing dupilumab and mepolizumab as add-on therapies in eosinophilic COPD—are derived from trial populations that do not reflect the multimorbid, frail, and polypharmacic older patients who constitute the majority of those presenting for COPD management in clinical practice. Extrapolating these findings to patients aged 75–85 years with cardiovascular comorbidities, reduced renal function, cognitive impairment, or nutritional compromise introduces substantial uncertainty regarding the magnitude and durability of treatment benefits, as well as the risk of adverse events not captured in trial populations. Furthermore, the logistical requirements of subcutaneous self-administration and stringent cold-chain management may pose additional practical barriers for older patients with reduced manual dexterity or cognitive decline. Real-World Evidence (RWE) generated from post-approval observational studies, electronic health records, and disease registries represents an essential complement to clinical trial data for bridging this evidence gap. RWE can capture the heterogeneous spectrum of older patients with COPD in routine clinical settings, including those systematically excluded from trials, and can provide valuable insights into treatment effectiveness, safety signals in the context of polypharmacy, and the role of age-related moderators in biologic treatment response. Physicians themselves increasingly recognize the importance of RWE for informing prescribing decisions in older patients for whom trial-derived evidence is limited or absent [94]. Moving forward, dedicated geriatric COPD studies—incorporating standardized assessments of frailty, functional status, cognitive function, and multimorbidity burden—as well as more inclusive trial designs with relaxed eligibility criteria, will be essential to ensure that the promise of precision biologic therapy is fully realized in the population that needs it most.

## 6. Future Perspectives

Advancing precision medicine within the older COPD demographic requires a paradigm shift away from chronological age toward parameters of biological age and functional reserve. Future clinical trial designs must transition to pragmatic frameworks with relaxed eligibility criteria that reflect real-world clinical practice, systematically enrolling individuals older than 75 and 85 years. To achieve external validity, future studies evaluating biologic agents in older populations should incorporate validated frailty instruments—such as the Clinical Frailty Scale (CFS) [95], the FRAIL Scale [96], or a formalized Frailty Index [97]—as prespecified stratification variables rather than exploratory post hoc metrics. Moreover, future precision protocols should evaluate a wider array of biological age markers, including sarcopenia indices [98], gait speed [99], physical resilience measures [73], and epigenetic aging biomarkers (such as DNA methylation clocks) [100,101,102] to characterize physiological vulnerability accurately. Crucially, clinical efficacy endpoints in geriatric biologic trials must expand beyond traditional respiratory parameters like FEV1 and annualized exacerbation rates. Future investigative programs must systematically assess multi-dimensional, patient-centered functional outcomes, including physical performance scores, mobility preservation, activities of daily living (ADLs), hospitalization-free survival, and the maintenance of functional independence. For the oldest segment of the population, the preservation of functional capacity and quality of life represents a primary therapeutic target that may carry greater clinical significance than minor incremental improvements in spirometric parameters.

### Limitations of the Current Evidence Base

The primary limitation of this review stems from the lack of primary literature specifically designed to evaluate biologic interventions in the oldest-old cohorts (≥75 and ≥80 years). The data synthesized herein are predominantly derived from trials where the average participant age was approximately 65 years, and where individuals with significant polypharmacy, severe renal impairment, unstable cardiovascular disease, or cognitive conditions were systematically excluded. Consequently, the direct generalizability of these findings to complex internal medicine and geriatric wards remains limited, necessitating caution when translating trial-derived safety and efficacy guidelines to frail older adults in routine clinical practice. This highlights the critical need for post-marketing real-world evidence (RWE) registries to monitor long-term safety and effectiveness in unselected populations.

## 7. Conclusions

**Figure 2:**
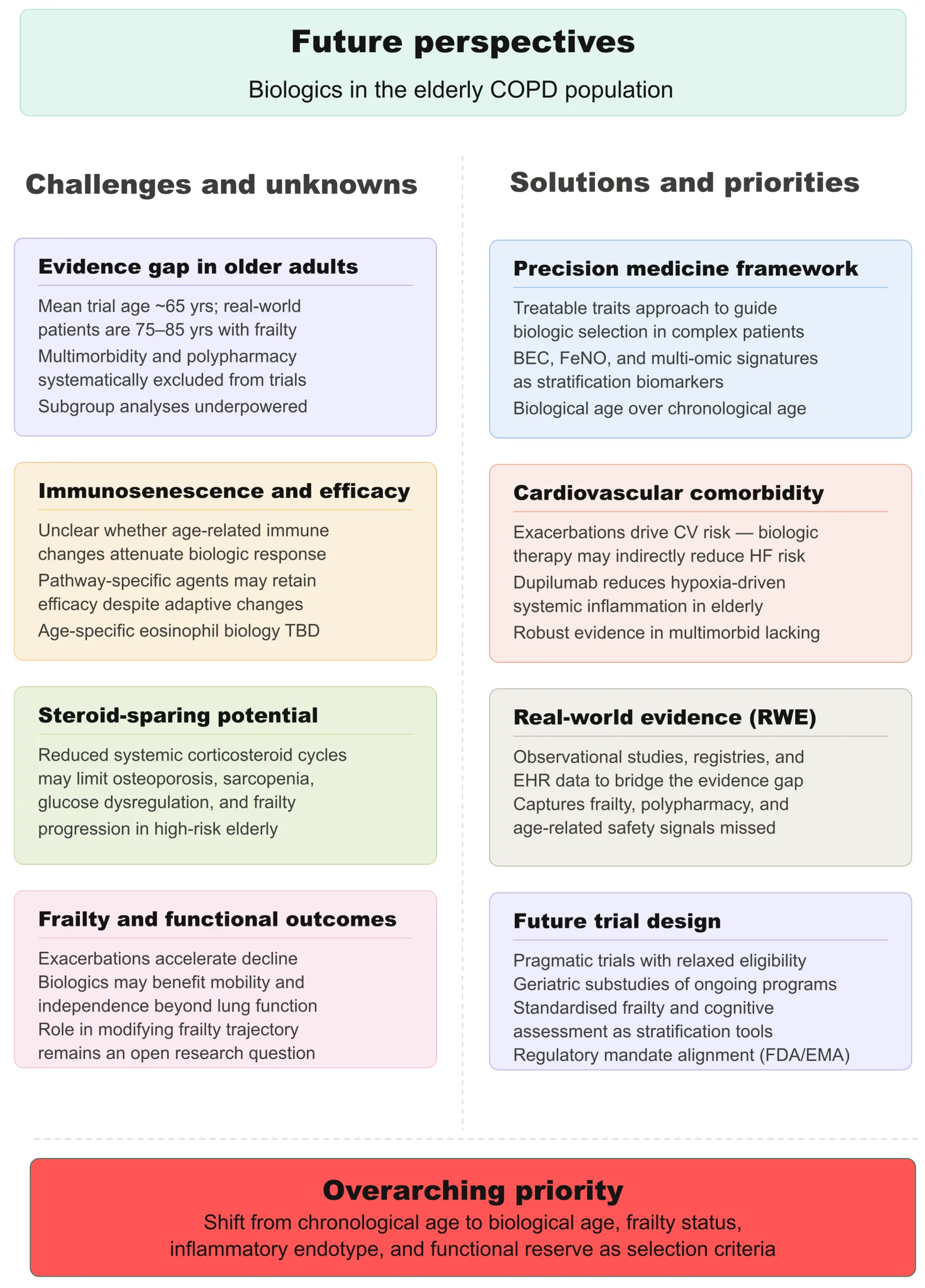
Future Perspectives in biologic treatment in COPD.

Biologics targeting type 2 inflammatory pathways represent a genuinely novel and mechanistically grounded therapeutic option for a subgroup of patients with COPD characterized by eosinophilic airway inflammation. The clinical trial evidence, culminating in the BOREAS and NOTUS trials and their pooled analysis, provides compelling support for dupilumab as an effective add-on therapy in patients with blood eosinophil counts ≥ 300 cells/μL and recurrent exacerbations. However, the translation of this evidence into the older COPD population—which constitutes the vast majority of real-world patients—requires careful consideration of the biological, clinical, and pharmacological dimensions uniquely associated with aging. It remains unclear whether immunosenescence may attenuate the therapeutic response to biologics in older adults. Since several biologic agents act on highly specific inflammatory pathways rather than inducing broad immunosuppression, it is conceivable that their efficacy may be preserved despite age-related alterations of adaptive immunity. Conversely, age-dependent changes in eosinophil biology, innate immune activation, and cytokine signaling may influence treatment responsiveness and require further investigation. Multimorbidity and polypharmacy introduce pharmacokinetic and pharmacodynamic complexities, and competing cardiovascular and metabolic risks must be integrated into individualized treatment decisions. Recurrent COPD exacerbations frequently accelerate functional decline and frailty progression in older adults. Therefore, the benefits of biologic therapies may extend beyond respiratory outcomes, potentially influencing mobility, independence, and quality of life. Whether biologics can modify the trajectory of frailty remains an important unanswered question. Beyond exacerbation reduction, biologics may offer an important steroid-sparing strategy in older patients. Reducing exposure to repeated systemic corticosteroid courses could potentially decrease the burden of steroid-related complications, including osteoporosis, sarcopenia, glucose dysregulation, neuropsychiatric effects, and frailty progression. The treatable traits framework, supported by robust biomarker stratification, offers a structured pathway for precision biologic prescribing in this population. Yet the absence of dedicated evidence in patients aged ≥75 years, particularly those with frailty and significant comorbidity, means that current clinical recommendations rest on a fragile empirical foundation when applied to the older. Chronological age alone should probably not be considered a limiting factor for biologic therapy. Future treatment algorithms may increasingly rely on biological age, frailty status, inflammatory endotyping, and functional reserve rather than chronological age itself (Figure 2). Addressing this gap will require a concerted effort from the research community, regulatory agencies, and pharmaceutical sponsors. Pragmatic clinical trials with broadened eligibility criteria, targeted geriatric substudies of existing biologic programs, and systematic collection of RWE from registry-based cohorts represent complementary and urgent priorities. Without such evidence, the potential of biologics to improve outcomes, reduce exacerbation burden, and enhance quality of life in the largest and most vulnerable segment of the COPD population will remain largely theoretical—an underexplored area in an era of otherwise rapidly advancing precision medicine.

## Figures and Tables

**Table 1:** Analysis of Geriatric Characteristics, Age Exclusion Criteria, and Comorbidities in Major RCTs on Biologics for COPD.

Study/Biologic	Enrollment Age	Excluded Comorbidities	Geriatric Strengths and Limitations
**BOREAS/NOTUS** (Dupilumab) [17,33]	40–80 years (BOREAS) 40–85 years (NOTUS) *Excluded: >80/85 years*	Other pulmonary diseases (e.g., pulmonary fibrosis, bronchiectasis, pulmonary hypertension). Systemic diseases associated with hypereosinophilia.	**Limitation:** Formal exclusion of the oldest-old. Real-life studies highlight that a large portion of the eligible population in clinical practice is >80 years old and would not meet RCT criteria [34].
**MATINEE** (Mepolizumab) [27]	≥40 years *No specified upper age limit*	Active lung cancer, tuberculosis, unstable liver diseases (cirrhosis, ascites, encephalopathy).	**Strength:** Included patients with concomitant cardiovascular risk (72% of the sample had CV risk factors and 25% had known cardiac disease) [27]. Highly representative of the typical older patient.
**ABRA** (Benralizumab) [31]	≥18 years *No specified upper age limit*	*Unstable* renal, hepatic, or cardiovascular failure. Hypercapnic respiratory failure. Life expectancy <6 months.	**Limitation:** The exclusion of patients with cardiovascular/renal instability and low life expectancy precludes the enrollment of the frailest geriatric patients or those receiving concurrent palliative care.
**COURSE** (Tezepelumab) [38]	40–80 years *Excluded: >80 years*	Asthma or overlap phenotype (ACO), and clinically relevant pulmonary diseases other than COPD.	**Limitation:** The strict age cut-off at 80 years prevents evaluating the efficacy of TSLP inhibition in the extreme senile population, which is frequently affected by irreversible remodeling [38].
**AERIFY-1/2** (Itepekimab) [39]	40–85 years *Excluded: >85 years*	Right heart failure, cor pulmonale, arrhythmias, uncontrolled hypertension. Malignancies in the past 5 years.	**Limitation:** Severe exclusion of recent neoplastic diseases and heart failure, which are highly prevalent comorbidities epidemiologically linked to advanced COPD in older patients [39].
**FRONTIER-4** (Tozorakimab) [40]	40–80 years *Excluded: >80 years*	Asthma [40].	**Limitation:** Once again, the arbitrary exclusion of subjects aged >80 years reduces the external validity of the results for geriatric and internal medicine wards.

## Data Availability

The original contributions presented in this study are included in the article. Further inquiries can be directed to the corresponding author.
